# Impaired dopamine metabolism in Parkinson’s disease pathogenesis

**DOI:** 10.1186/s13024-019-0332-6

**Published:** 2019-08-20

**Authors:** Anna Masato, Nicoletta Plotegher, Daniela Boassa, Luigi Bubacco

**Affiliations:** 1 Department of Biology, University of Padova, Padova, Italy; 2 Department of Neurosciences, and National Center for Microscopy and Imaging Research, University of California San Diego, La Jolla, CA USA

**Keywords:** Parkinson’s disease, Selective vulnerability, Dopamine, DOPAL, αSynuclein, Aldehyde dehydrogenase

## Abstract

A full understanding of Parkinson’s Disease etiopathogenesis and of the causes of the preferential vulnerability of nigrostriatal dopaminergic neurons is still an unsolved puzzle. A multiple-hit hypothesis has been proposed, which may explain the convergence of familial, environmental and idiopathic forms of the disease. Among the various determinants of the degeneration of the neurons in *Substantia Nigra pars compacta*, in this review we will focus on the endotoxicity associated to dopamine dyshomeostasis. In particular, we will discuss the relevance of the reactive dopamine metabolite 3,4-dihydroxyphenylacetaldehyde (DOPAL) in the catechol-induced neurotoxicity. Indeed, the synergy between the catechol and the aldehyde moieties of DOPAL exacerbates its reactivity, resulting in modification of functional protein residues, protein aggregation, oxidative stress and cell death. Interestingly, αSynuclein, whose altered proteostasis is a recurrent element in Parkinson’s Disease pathology, is considered a preferential target of DOPAL modification. DOPAL triggers αSynuclein oligomerization leading to synapse physiology impairment. Several factors can be responsible for DOPAL accumulation at the pre-synaptic terminals, i.e. dopamine leakage from synaptic vesicles, increased rate of dopamine conversion to DOPAL by upregulated monoamine oxidase and decreased DOPAL degradation by aldehyde dehydrogenases. Various studies report the decreased expression and activity of aldehyde dehydrogenases in parkinsonian brains, as well as genetic variants associated to increased risk in developing the pathology. Thus, we discuss how the deregulation of these enzymes might be considered a contributing element in the pathogenesis of Parkinson’s Disease or a down-stream effect. Finally, we propose that a better understanding of the impaired dopamine metabolism in Parkinson’s Disease would allow a more refined patients stratification and the design of more targeted and successful therapeutic strategies.

## Background

Parkinson’s Disease (PD) is an age-related, severe neurodegenerative movement disorder. The pathology affects about 1% of the population over 65 years old and more than 4–5% over 80, being the latter the current average life expectancy in the European Community [1, 2]. Clinical PD is a multi-factorial pathology and most of the cases are classified as sporadic with an undefined aetiology, while only 5–10% of cases have genetic causes. At the histological level, the progressive neuronal loss corresponds to the accumulation of proteinaceous intra-cytoplasmic inclusions, named Lewy Bodies (LBs), in which amyloid fibrils of the presynaptic protein αSynuclein (αSyn) are the main constituent [3].

It has been proposed that, during the development of the pathology, neurodegeneration gradually interests different regions of the brain although it mostly affects the nigrostriatal circuits in the midbrain. This results in the typical motor symptoms, as the nigrostriatal pathway is involved in voluntary movement coordination of the body. Indeed, after the loss of more than 80% of the dopaminergic neurons in the *Substantia Nigra pars compacta* (SNpc), parkinsonian syndrome manifests with tremor at rest, rigidity, slowness or absence of voluntary movement, postural instability and freezing [4, 5]. This view however, is still object of debate, as it has been recently challenged by Engelender and Isacson, who argued that the observed ascending progression of the disease may result from a combination of a diverse vulnerability of Central Nervous System and Peripheral Nervous System, as well as different “functional reserve” of the neurons involved [6].

The identification of causative factors responsible for the preferential vulnerability of dopaminergic neurons of SNpc is still an unsolved quest in PD research and its purported molecular determinants have been recently reviewed by Brichta and Greengard [7]. The remaining challenge is still in understanding why mutations in various proteins with different or unclear physiological functions converge to similar pathological phenotypes, which are also observed in idiopathic PD cases [8]. Conversely, familial, environmental and idiopathic PD forms present some differences from both the histopathological and clinical point of view. For example, PD patients carrying *Parkin*, *Pink1* or *Lrrk2* mutation do not always present LBs [8, 9]. Moreover, patients differ in terms of age of onset, disease severity, progression of the neurodegeneration and type of symptoms (motor and non-motor).

On this ground, a multiple-hit hypothesis for PD pathogenesis has been put forward [10, 11]. According to this hypothesis, several risk factors, both genetic and environmental, concomitantly affect neuronal homeostasis resulting in progressive neurodegeneration [10, 11]. This hypothesis may explain both similarities and divergences in the different PD forms and it would allow patient stratification. As Surmeier and colleagues recently reviewed, the analysis of morphological, functional and molecular peculiarities of the SNpc dopaminergic neurons is starting to shed some light on their selective vulnerability in PD [5, 12]. As main features, this neuronal population presents an intrinsic low calcium buffering capacity and the ability to perform pace-making activity [13]. Moreover, the dopaminergic neurons carry the machinery to metabolize and catabolize dopamine (DA), the neurotransmitter synthetized and secreted in the nigrostriatal pathway.

Among these important aspects (which may be not mutually exclusive in determining dopaminergic neurons vulnerability), our interest here will mainly focus on the role of DA metabolism and catabolism in PD etiopathogenesis. Indeed, the endotoxicity derived from increasing DA levels, DA oxidation and its reactive catabolites, is recognised as one of the major causes of oxidative stress in PD [14–17]. Interestingly, several PD-related proteins appeared to participate in the modulation of the dopaminergic pathway in health and disease [18, 19]. Therewithal, αSyn, whose altered proteostasis is primarly involved in molecular mechanisms responsible for neuronal death, has been highlighted as preferential target of DA-related neurotoxicity [20, 21].

In the last decades, the concept that a dyshomeostasis of catechol amines may lead to endotoxicity has been extended to DA catabolites, as many studies revealed impaired DA metabolites in PD models and autoptic samples [22]. Among the several metabolites monitored, attention was addressed on 3,4-dihydroxyphenylacetaldehyde (DOPAL), a toxic DA catabolite. In this review, we aim to discuss evidence that support DOPAL involvement in the pathogenesis of PD, its potential synergy in αSyn-induced pathology and whether DOPAL toxicity might contribute to rationalize the deleterious effects on nigral neurons that have been referred solely to DA.

## 3,4-Dihydroxyphenylacetaldehyde: a relevant player in dopaminergic neuron degeneration

**Fig. 1:**
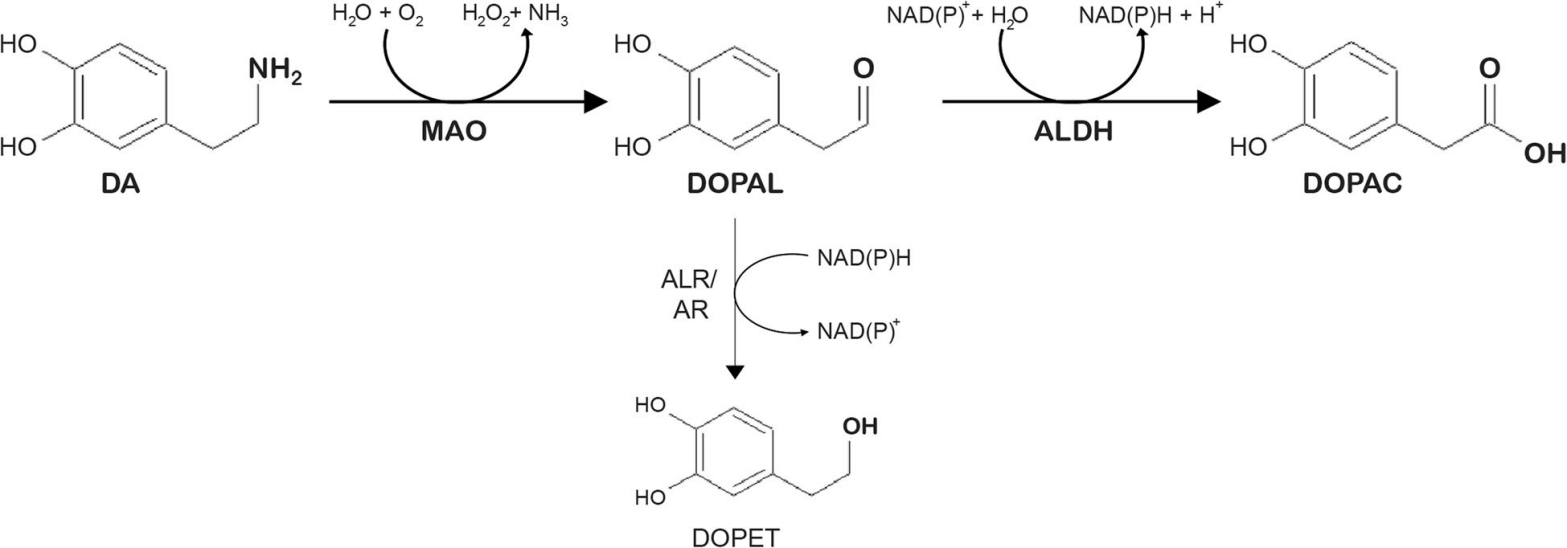
Dopamine catabolism. In dopaminergic neurons, DA catabolism starts with deamination by MAO to generate DOPAL. The aldehyde moiety is then converted to the carboxyl group of DOPAC by ALDHs. A smaller fraction of DOPAL aldehyde is converted to the hydroxyl group of DOPET by ALR/ARs (thinner arrow)

DA levels within SNpc neurons are strictly regulated, as an equilibrium among synthesis, synaptic vesicle loading, uptake from the extracellular space and catabolic degradation [16]. As showed in Fig. 1, DA catabolism starts with the oxidative deamination, a reaction mediated by the mitochondrial monoamine oxidase (MAO), which also generates H_2_O_2_ and ammonia. The resulting product, DOPAL, is further metabolized either to 3,4-dihydroxyphenylacetic acid (DOPAC) or 3,4-dihydroxyphenylethanol (DOPET) by aldehyde dehydrogenase (ALDH) or by aldehyde/aldose reductase (ALR/AR), respectively.

Although DOPAL is a physiological intermediate in DA catabolism, it resulted to be an endogenous neurotoxin [23]. Being an aldehyde, DOPAL is a very electrophilic molecule, prone to induce covalent modification of nucleophile functional groups in the cytoplasmic milieu [24]. DOPAL concentration in SNpc dopaminergic neurons has been estimated to be around 2–3 μM, a level compatible with the affinity reported for the DOPAL detoxifying enzymes previously mentioned (0.4–1 μM for ALDHs) [24]. Concentrations higher than physiological (> 6 μM) have been described as a threshold for cytotoxic effects in various cell lines [24]. Thereafter, the work of Burke et al. in 2003 provided substantial evidence of DOPAL neurotoxicity in vivo [25]. DOPAL injection in rat *nigral* dopaminergic neurons resulted in detrimental neuronal loss, more pronounced than that induced by administration of DA or its metabolites (DOPAC, DOPET, HVA). More recently, a post mortem study on sporadic PD patients’ brains revealed DOPAL build-up relative to DA in the putamen of PD subjects compared to healthy controls [26]. The levels of DA and its catabolites was determined by High Pressure Liquid Chromatographic separation coupled to Electro-Chemical Detection (HPLC-ECD) [27–29]. This technique, which is considered the ‘gold standard’ for catechols quantification in cells and tissues, allows singling out DOPAL from other catecholamines, based on its unique electro-chemical properties. Using the same technique, other correlated studies also reported decreased DOPAC:DOPAL ratio in PD, together with lowered vesicular sequestration of DA through the vesicular monoamine transporter type-2 (VMAT-2) [26, 27, 30]. Moreover, a decreased DOPAC content in cerebrospinal fluid (CSF) from samples of PD patients was measured, combined by 5-S-cysteinyl-DA/DOPAC ratios averaged more than twice compared to controls [31]. In this frame, these seminal results prompted the formulation of the *Catecholaldehyde hypothesis*, which underscores the key role of DOPAL in the molecular mechanisms responsible for SNpc degeneration in PD [23, 25, 32–35].

**Fig. 2:**
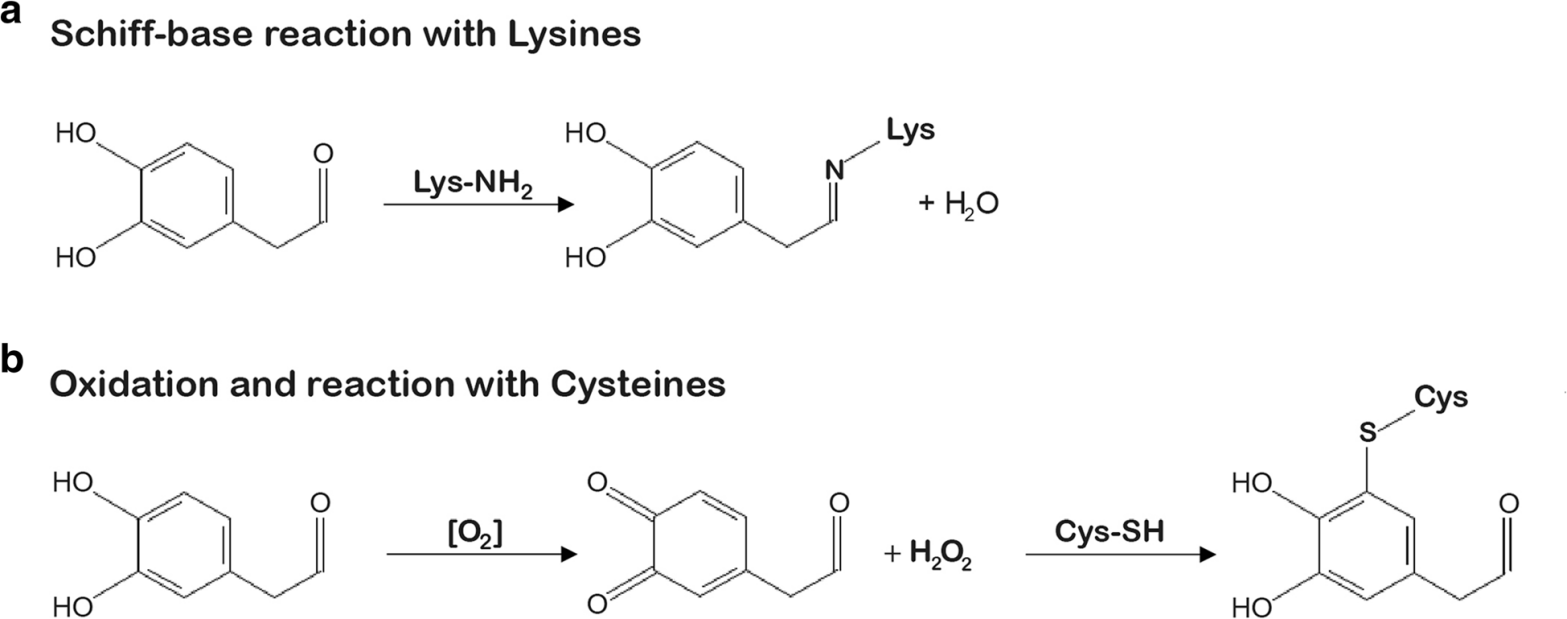
DOPAL reactivity and reported neurotoxic molecular mechanisms. DOPAL reactivity is due to both the aldehyde and the catechol moiety, respectively resulting in covalent modification of primary amines and thiols (i.e. lysine and cysteine residues of proteins) [36–38]. **a** DOPAL addiction to lysines is the result of a Schiff-base reaction between the aldehyde and the primary amine of the lysine’s lateral chain, with the release of a molecule of water. **b** In oxidative conditions, the catechol group has the tendency to auto-oxidation, with production of quinones and oxygen radical species [39]. Also, the oxidized cathecol is reactive towards the thiols of cysteines

DOPAL is a highly reactive molecule, which presents two functional groups that may account for its toxicity. These are the aldehyde and catechol moieties, which can both contribute to DOPAL reactivity toward proteins (Fig. 2). The first one targets mainly primary amines and the second thiols [36]. Of interest, the two moieties do not act independently of each other, in fact the oxidation of the catechol ring enhances the Schiff base reaction between the aldehyde moiety of DOPAL and primary amines [37]. Also, the oxidation of the catechol is required for the addiction of thiols to the aromatic ring. This implies that DOPAL is prone to covalently modify amino acid residues i.e. lysines and cysteines.

As mentioned above, the HPLC-ECD is the most reliable method to quantify the concentrations of catechols in solution. However, this method does not allow to measure the fraction of DOPAL that is bound to proteins. So far, the detection of catechol-modified proteins from cell lysates has been performed by SDS-Page followed by the staining with nitroblue tetrazolium (NBT), a redox-cycling dye for the detection of catechol adducts [34]. Alternatively, the protein pull-down assay with aminophenylboronic acid (APBA) resin allows the isolation of catechol-modified proteins from cell lysates [40, 41], with the caveat that it also binds glycosylated proteins. More recently, the near Infrared Fluorescence (nIRF) scanning was applied to the detection and quantification of o-quinones in cells and tissues, as well as proteins modified by oxidized catechols [18, 20, 42]. This method relies on the ability of quinones to emit a fluorescence signal after excitation at 700 nm upon stimulation at 685 nm [43]. Anyway, all these other techniques are way less sensitive than the HPLC-ECD, leaving a rigorous DOPAL quantification in biological samples a challenging task. As a consequence, the development of tools aimed to precisely quantify catechol levels and DOPAL-modified macromolecules in both experimental models and patients’ samples is needed.

**Fig. 3:**
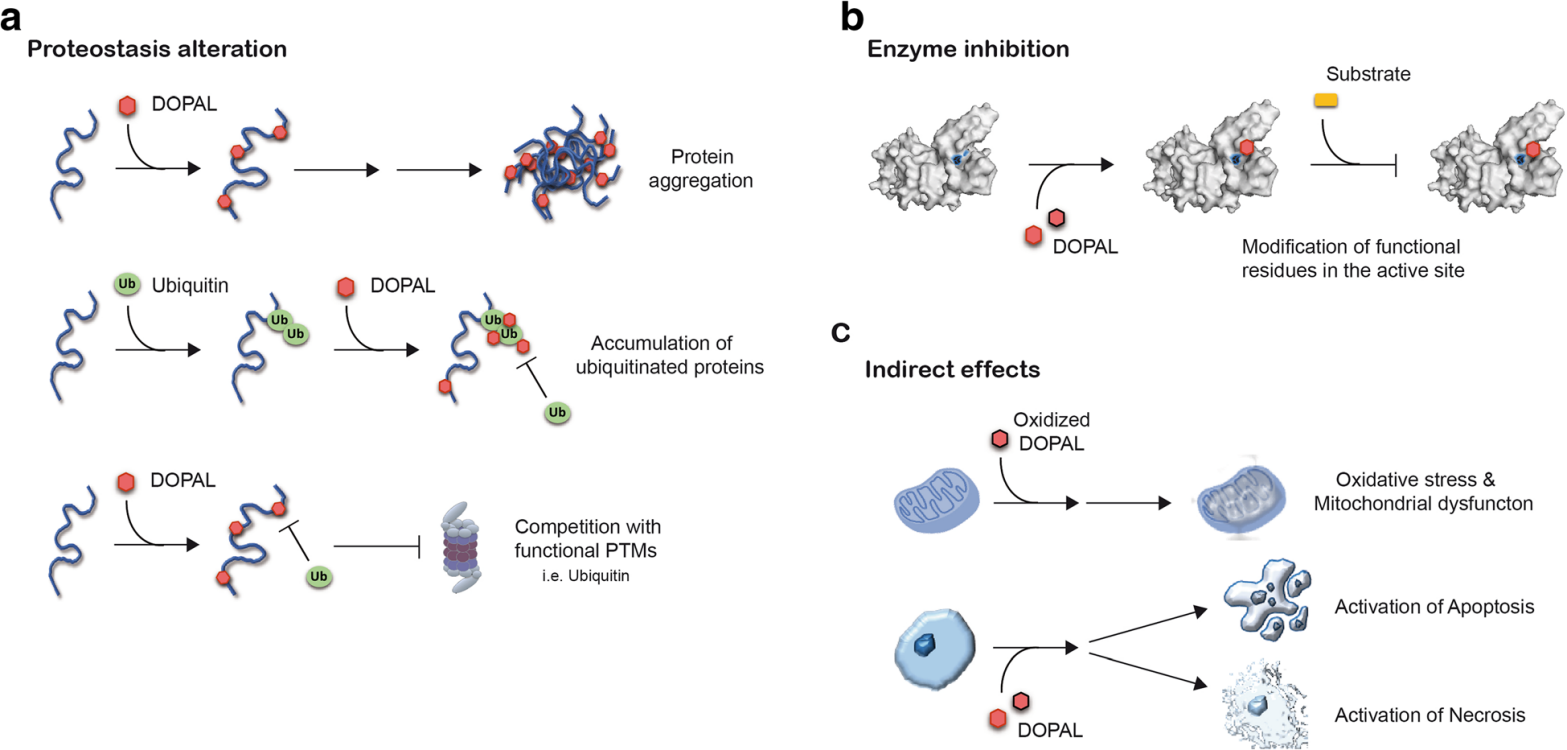
DOPAL reported neurotoxic molecular mechanisms. DOPAL build-up in SNpc dopaminergic neurons triggers multiple neurotoxic mechanisms: **a** alteration of neuronal proteostasis, in terms of protein aggregation [34, 36, 38, 41, 44], competition with functional post-translational modifications (PTMs, i.e. ubiquitination, SUMOylation, acetylation) and accumulation of ubiquitinated proteins [42, 45]; **b** enzyme inhibition (PDB: 4i1f, in the figure) [46–48]; **c** indirect effects, which imply oxidative stress [39], mitochondrial dysfunction [17, 49–51], activation of necrotic and apoptotic pathways [23, 24, 33]

DOPAL reactivity and accumulation in cells are believed to be detrimental to neurons and possibly responsible of different neurotoxic mechanisms. These were ascribed to DOPAL alone or to DOPAL-modified molecules, as summarized in Fig. 3.

### DOPAL-induced proteostasis alteration (Fig. 3a): effects on protein aggregation, on functional lysines and competition with other post-translational modifications

The high reactivity of both functional groups of DOPAL results in protein cross-linking which leads to protein aggregation. This was demonstrated by several in vitro studies, in which DOPAL was incubated with Glyceraldehyde-3-phosphate-dehydrogenase (GAPDH) and Bovine Serum Albumin (BSA) as model proteins [34, 36]. More interestingly, DOPAL was shown to trigger αSyn oligomerization to generate SDS-resistant high molecular weight species to whom pathological relevance in PD has been attributed [38, 41, 42, 44]. This issue will be extensively discussed in the following paragraph “Linking the *Catecholaldehyde Hypothesis* to αSyn-induced pathology”.

Lysine residues are often subjected to post-translational modifications (i.e. ubiquitination, SUMOylation, acetylation) that are important in regulating protein clearance, protein subcellular localization, protein-protein interactions and protein secretion through extra-cellular vesicles [45]. Of note, ubiquitin itself possesses seven functional lysines, through which poly-ubiquitin chains are synthetized to target proteins in different cellular compartments. It has been recently demonstrated that, in vitro, DOPAL modifies ubiquitin lysines and promotes ubiquitin oligomerization [42]. In the same work, DOPAL treatment on PC-12 cells resulted in accumulation of ubiquitinated proteins [42]. In this frame, more needs to be done to explore a potential scenario in which the chemical modification by DOPAL of lysine-rich proteins in neurons, would affect not only their proper functions but also their capability to be tagged by ubiquitin. As a consequence, dysfunctional DOPAL-modified proteins that should be targeted for clearance would end up in an aberrant accumulation because of DOPAL competition for their ubiquitination sites or the impairment of the ubiquitination pathway itself.

Along the same line, not only ubiquitin and the ubiquitination pathway, but also small ubiquitin-like modifier (SUMO) proteins may be the targets for DOPAL modifications. Four SUMO paralogues have been identified in humans (SUMO-1 to SUMO-4) and similarly to ubiquitin they present a large number of lysines in their sequence (for example, 11 lysines out of 101 amino acids in hSUMO-1). SUMOs substrates, SUMOs conjugation machinery and the specificity of paralogues towards diverse proteins are still object of extensive research. However, it is known that SUMOylation can act on several cellular processes as transcription and protein localization, by regulating protein-protein interaction and substrate conformational changes [52]. For instance, in neurons, SUMOylation of the glutamate receptor subunit 6 is responsible for the endocytosis of the receptor at the plasma membrane [53]. Limited information about the role of SUMOs in PD is available, but it was shown that this pathway is crucial for cellular function and survival. In fact, when Ubc9, which is required for conjugation of SUMO proteins to their substrate, is depleted, cells present nuclear abnormalities and undergo apoptosis [54]. Coherently, SUMOylation was reported to mediate αSyn sorting into lumen of vesicles and attenuate αSyn aggregation and toxicity [55–57]. It is then feasible to reason that covalent modifications to SUMO lysines due to DOPAL accumulation may reduce the amount of SUMOs available for the tight regulation of essential cellular processes. At the same time, DOPAL-modified lysines on the substrate proteins would affect the possibility for them to be SUMOylated.

Lysines modification by DOPAL may also impact on another relevant pathway for the regulation of different cellular functions, i.e. lysines acetylation. This process relies on acetyl-coenzyme A as the acetyl group donor and it was shown to regulate transcription factors, molecular chaperones, effectors and cytoskeletal proteins [58]. Many of these processes are crucial for the proper functions of the neurons. For example, it was shown that lysines acetylation is relevant in the turnover of huntingtin, a protein whose mutations cause its aggregation and are the cause of the incurable neurodegenerative disorder Huntington’s disease. Huntingtin acetylation alters the protein aggregation propensity [59] and regulates protein targeting for auto-phagosomal degradation [60]. This may also be of relevance for PD, being that αSyn is acetylated at its lysine residues [61], but the signalling pathways that are regulated by this PTM on αSyn are still unclear. Overall, if DOPAL modification on protein substrates competes for acetylation signalling, many crucial cellular processes may be affected.

Interestingly, it has been observed that there is a cross-talk among ubiquitination, SUMOylation and lysines acetylation pathways and a co-regulation of substrate proteins exist [52]. If DOPAL-induced changes in the level of one of these PTMs for a certain substrate alter also one of the other pathways, the already complex picture described so far may be further convoluted and would deserve careful evaluation.

### DOPAL-induced enzyme inhibition (Fig. 3b)

Protein modification by DOPAL has deleterious outcomes also for enzyme activity. In fact, any enzyme with an accessible functional cysteine or lysine in the active site could be susceptible to inactivation by DOPAL, with important upshots on the metabolic pathways of interest. For instance, a proteomic study on PC6–3 cells identified tyrosine hydroxylase (TH) as target of DOPAL [62]. Administration of DOPAL at physiologically relevant concentration (5-50 μM in the cell medium) resulted in 80–95% of TH activity inhibition, as assessed by TH purification from cells followed by HPLC quantification of L-DOPA production. The authors speculated that DOPAL induces rearrangement of enzyme conformation, by modifying lysine residues that are present within or in close proximity of the active site [46]. Since TH activity is a rate-limiting step in DA synthesis from tyrosine, DOPAL-dependent TH inhibition would indirectly exacerbate the depletion of DA release in nigrostriatal circuits and parkinsonian syndrome. More recently, DOPAL appeared to cause inhibition of GAPDH activity [47]. Also, in this paradigm, both the catechol oxidation and the aldehyde moiety were required for cysteines and lysines modification. An analogous effect has been shown for DA (at least for the Cysteine residues), which was reported to modify and functionally inhibit parkin, an E3 ubiquitin ligase with genetic correlation to early-onset of PD (PARK2 locus) [48]. It is plausible to think that inactivation of parkin through catechol-cysteine adducts might involve also the catechol moiety of DOPAL, that has been reported to be even more reactive than DA [25, 36, 49].

### Downstream effects of DOPAL accumulation: oxidative stress, mitochondrial dysfunction and cell death (Fig. 3c)

Several studies based on cellular model systems confirmed time- and concentration-dependence of DOPAL cytotoxicity [24]. The direct participation of DOPAL in oxidative stress has been investigated, as DOPAL can generate radical species, i.e. hydroxyl radical, in the presence of H_2_O_2_ [35]. DOPAL catechol group has a propensity to auto-oxidise to semiquinone radicals and ortho-quinones similar to DA [39]. The resulting radical oxygen species (ROS) production (Fig. 2) is expected to exacerbate the oxidative stress in neurons, leading to DNA damages, protein cross-linking and lipid peroxidation. Interestingly, cyclooxygenase-2 (COX-2), an enzyme involved in neuroinflammation and up-regulated in the SNpc of parkinsonian brains [39], was reported to catalyze DA oxidation. In the work by Anderson and colleagues, even DOPAL was shown to be a substrate of COX-2, accelerating the oxidation of DOPAL catechol as for DA. Thus, these results reiterated a connection among different aspects of PD: endotoxic catecholamines, oxidative stress and neuroinflammation, together with the potential relevance of antioxidant effectors [63]. Superoxide dismutase (SOD) may be of interest, as it efficiently clears superoxide anion by dismutation into molecular oxygen and hydrogen peroxide, removing the oxidative agent from the cellular milieu. Indeed, a recent work demonstrated that, at least in vitro, SOD1 is able to prevent lysines modification by DOPAL and associated protein cross-linking, acting as enzymatic antioxidant [64]. Other in vitro studies revealed that antioxidants agents such as N-acetylcysteine, glutathione and ascorbic acid could effectively modulate the level of DOPAL-modified proteins in a dose-dependent manner [37, 42].

A further analogy with DA is that also DOPAL quinones could covalently modify mitochondrial protein, possibly affecting mitochondrial physiology [50]. In the work by Kristal et al., isolated mitochondria from mouse liver were exposed to DOPAL resulting in an increased opening of the mitochondrial permeability transition pore (mPTP) at concentrations close to physiological ones (0.125–8 μM) [49]. Later studies reported that DA oxidation to quinones (DAQs) induced mitochondria swelling and reduced respiratory activity, suggesting the induction of the mPTP opening [17]. An analogous effect was ascribed to DAQs derived from enzymatic oxidation of DA, specifically addressing the modulation of mPTP opening to DAQs [51]. As a consequence, both DA and DOPAL-derived quinones could be responsible for the activation of the apoptotic pathway. On the other hand, DOPAL-induced decreased cell viability was assessed by measuring Lactate Dehydrogenase (LDH) release in the extra-cellular space, which is an accepted indication of necrosis [23, 33].

## Linking the *Catecholaldehyde hypothesis* to αSynuclein-induced pathology

Since the identification of αSyn in LBs 20 years ago and its association with some familial forms of PD, the relevance of αSyn in the pathogenesis of PD has been widely investigated [3]. Particular interest has been given to the downstream effects of αSyn aggregates accumulation on neuronal homeostasis, leading to the notion that they could impair many cellular pathways and undermine organelles integrity [65, 66]. In this frame, several research groups focused their attention on the interplay between DOPAL and αSyn. Starting from the observed reactivity of DOPAL aldehyde against primary amines of lysine residues, the aim has been to investigate whether DOPAL modification on αSyn would affect both its aggregation properties and its proteostasis. Indeed, αSyn might be considered a preferential target of DOPAL for at least three reasons [45]. First, lysine accounts for 10.7% of αSyn sequence, which is higher than the average value (around 5%) of the lysine fraction in synaptic proteins [45]. Most of the lysines in αSyn sequence are within the amino acid repeats containing the consensus motif KTKEGV, which drives the transition to the alpha-helical conformation of αSyn N-terminus and the association to synaptic vesicles membranes [67]. Second, αSyn represents the 0.5–1% of the total soluble proteins of the brain, reaching a concentration up to 40 μM in pre-synaptic terminals of neurons, where it exerts its physiological function in association with synaptic vesicles membranes [68–70]. Consistently, DOPAL is mainly generated at pre-synaptic site, where MAO on the outer mitochondrial membrane quickly clears cytosolic DA in case of anomalous dyshomeostasis [71]. The third reason that points to αSyn as preferential target for DOPAL, is that when in the soluble monomeric state, it is an intrinsically disordered protein with good accessibility to all its lysine residues, making any potential chemical modification more likely.

A pivotal study by Burke et al. in 2008 demonstrated that in vitro DOPAL incubation with αSyn monomers triggers a dose-dependent protein aggregation. Similarly, SDS-resistant aggregates of αSyn were detected by Western Blot in lysates from SH-SY5Y cells after administration of DOPAL in the medium. The process was observed also in vivo upon direct DOPAL injection into rat SNpc, which resulted in dopaminergic neuron loss and accumulation of αSyn high molecular weight species [44]. Since then, other groups provided further insights into the DOPAL-dependent αSyn aggregation process. Inhibition of DA uptake into synaptic vesicles by reserpine administration to dopaminergic PC12 cells, induced DA cytosolic build-up with consequent cytotoxic accumulation of DOPAL and induction of αSyn oligomerization [72]. Furthermore, redox active metal ions i.e. Cu, Fe, Mn, whose levels are increased in parkinsonian SNpc [73], were shown to accelerate DOPAL-induced αSyn oligomerization in PC12 cells [74]. On the same ground, in vitro assays revealed a modulating effect of N-terminal acetylation and familial mutations (A30P, A53T, E46K, G51D, H50Q) on DOPAL-induced αSyn oligomerization [75].

More studies were conducted by Follmer and colleagues in 2015 and by our group in 2017 [38, 41]. The former authors identified by mass spectrometry the lysine residues of αSyn that seem to be preferentially modified by DOPAL upon in vitro incubation. These modification sites were mainly located at the lysine-enriched N-terminus of αSyn. Coherently, our experiments revealed overlapping results in vitro, but with the observation of additional modification sites involving lysine residues in the C-terminal domain upon formation of the αSyn-DOPAL adduct within cells. DOPAL modification of αSyn lysines dramatically alters αSyn biochemical and biophysical properties, increasing its hydrophobicity at the expense of the positive charges. Moreover, in vitro analysis revealed that DOPAL triggers αSyn aggregation leading to annular-shaped off-pathway oligomers, which do not convert to fibrils [41].

**Fig. 4:**
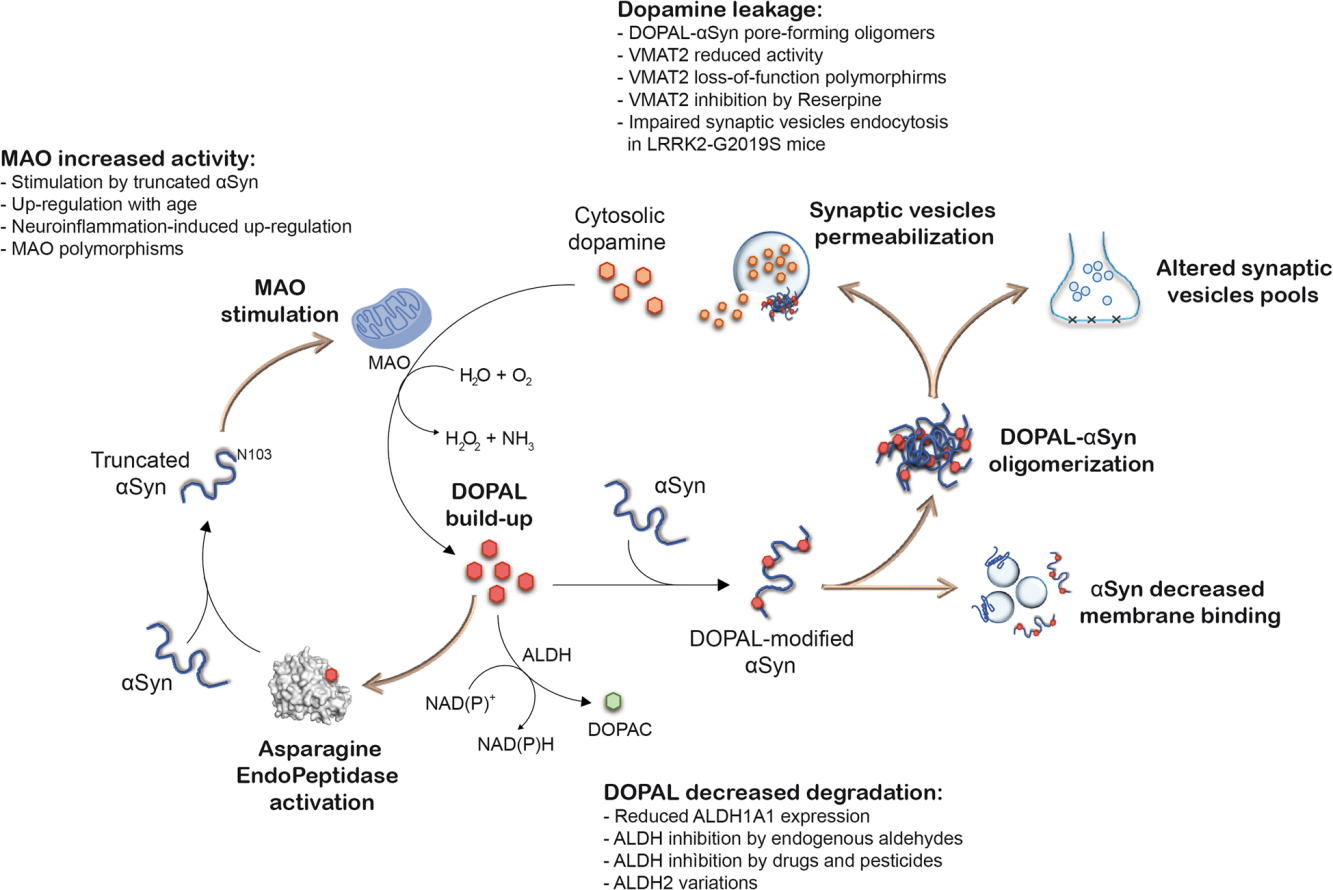
Potential interplay between DOPAL and αSynuclein at pre-synaptic terminals and determinants of DOPAL accumulation. DOPAL accumulation at the pre-synaptic terminals covalently modifies αSyn lysines, reducing αSyn affinity for membrane binding and resulting in synaptic vesicles pools redistribution [38, 41]. αSyn-DOPAL oligomers accumulate and permeabilize synaptic vesicles membrane [41], leading to cytosolic DA release, which is further metabolized into DOPAL by MAO. Also, DOPAL activates AEP (PDB: 4aw9, in the figure), which cleaves αSyn at N103 [76]. Truncated αSyn is more prone to aggregation and stimulates MAO activity. Hence, the result is a positive loop that self amplifies, leading to αSyn aggregation and synapse degeneration. In the figure, the black thin arrows indicate the chemical reactions, while the thicker ones highlight the cellular processes. Among the factors that could lead to DOPAL build-up, the critical hubs are the dysfunction of DA storage in synaptic vesicles, increased rate of DA degradation by MAO and decreased DOPAL detoxification by ALDHs. For each point, the evidences are listed in the figure

A coherent mechanism can be proposed (as illustrated in Fig. 4), based on the observed functional effects of the reaction between αSyn and DOPAL on synaptic vesicles and accounting for the degeneration of the dopaminergic synapse. An increased level of DOPAL at presynaptic site promotes the covalent modification of αSyn. DOPAL-αSyn monomers exhibit reduced affinity for membrane binding [38], shifting the equilibrium toward an increased fraction of cytoplasmic αSyn-DOPAL, thus exacerbating αSyn aggregation. A further consequence is that alterations in the levels of the membrane-bound fraction of αSyn dramatically impair its synaptic physiological function, as αSyn modulates both vesicles clustering and exocytotic events [41, 77–79]. In addition, we proposed that DOPAL-modified αSyn oligomers might be able to form aggregated oligomers that permeabilize the membrane of synaptic vesicles, thus inducing the release of DA in the cytoplasm, that will be in turn metabolized by MAO into more DOPAL [41]. Taken together, all these events would establish a toxicity self-amplifying loop, which leads to synaptic degeneration. In addition, a very recent study highlighted a potential role for the activity of asparagine endopeptidase (AEP). AEP is reported to be highly activated in PD patients’ brain where it can generate a truncated form of αSyn [80]. Interestingly, the resulting N103-truncated αSyn was shown to stimulate MAO-B activity, leading to increased rate of DOPAL production. Not only, DOPAL itself was observed to interact with and stimulate AEP, establishing an additional trail in the noxious cycle described above [76].

**Fig. 5:**
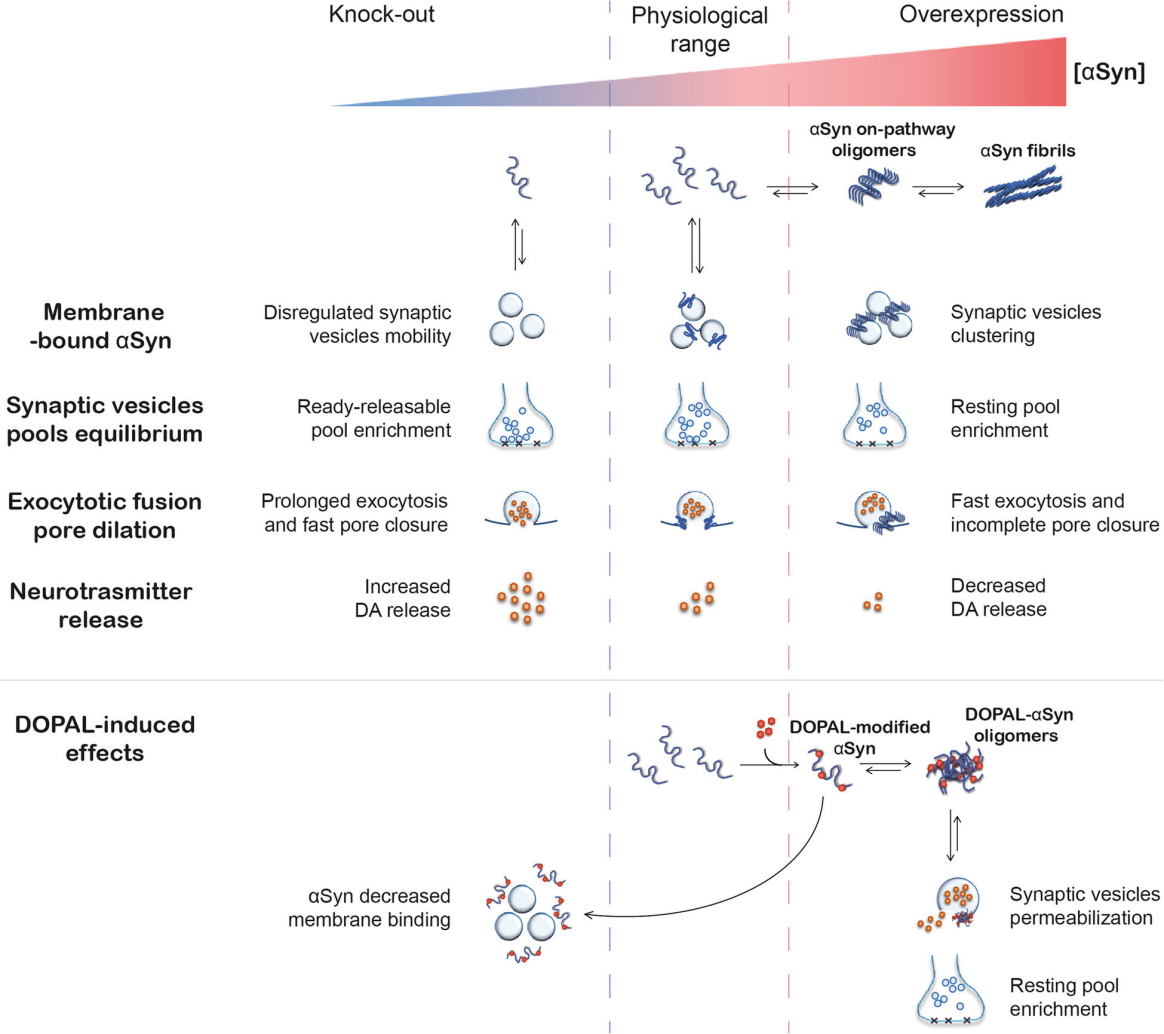
Effects of αSynuclein dyshomeostasis on synapse functionality. Under physiological conditions, αSyn ensures the correct balance of DA release in the striatum by binding to synaptic vesicles membrane, regulating vesicles mobility and the exocytotic events. However, upon αSyn dyshomeostasis, which includes both αSyn accumulation or its absence, the synaptic vesicles distribution among the different pools and the neurotransmitter release are altered, as demonstrated in the Syn-TKO and the αSyn-OVX mouse models [77, 79, 82–84]. Conversely, the DOPAL-αSyn interplay presents an additional level of complexity. Indeed, DOPAL modification of αSyn lysines hinders its association to synaptic vesicles membrane, mimicking a KO-like phenotype [38]. At the same time, DOPAL triggers αSyn aggregation in off-pathway pore-forming oligomers, resulting in synaptic vesicles permeabilization [41]. Furthermore, DOPAL build-up induces synaptic vesicles clustering of the resting pool, resembling the αSyn-overexpressing scenario [41]

The functional implications of the DOPAL-induced αSyn dyshomeostasis at the synapses merit careful consideration. Evidence from the literature suggests a role of αSyn in modulating synaptic vesicles clustering, SNARE complex assembly, vesicles docking at the active zone and opening of the exocytotic fusion pore [77, 79, 81]. Importantly, both the triple knock-out mouse lacking the three synuclein isoforms (alpha, beta and gamma – Syn-TKO) and the αSyn-overexpressing mouse model (αSyn-OVX) display alterations in the synapse architecture and neurotransmitter release. In particular, the αSyn overexpression results in impaired vesicles clustering with reduced vesicles density at the active zone; fast and incomplete exocytotic fusion pore dilation and pore closure; consequent decreased DA release in the striatum, weakening the nigrostriatal pathway [77, 79, 82]. Conversely, in the Syn-TKO mouse, more pronounced DA release was detected, potentially due to an accumulation of synaptic vesicles in the ready-releasable pool, prolonged exocytotic fusion pore dilation, faster neurotransmitter release and pore closure [79, 83, 84]. Taken together, these observations lead to the concept that although αSyn is not a limiting factor in the synapse activity, it is essential for maintaining the proper balance in neurotransmitter release and synaptic vesicles distribution. On this ground, since DOPAL modification of αSyn prevents its association to synaptic vesicle membrane, it could be considered a KO-like phenotype [38]. Yet, the observed DOPAL-induced synaptic vesicles redistribution, from ready-releasable pool to resting pool, together with αSyn accumulation may lean towards an overexpression-like scenario [41]. However, as DOPAL modification of αSyn lysines also triggers its aggregation, it may affect both synaptic vesicles mobility, docking, exocytosis and endocytosis. In addition, the pore-forming activity of the DOPAL-αSyn oligomers increases the complexity of the scenario, placing the DOPAL-αSyn interplay on a different level beyond the one where the Syn-TKO and the αSyn-OVX models are set, as exemplified in Fig. 5. Hence, future investigations will be of interest to better define the impact of DOPAL on αSyn homeostasis in the light of the synaptic mechanisms that αSyn influences.

Considering the neurotoxic potential of DOPAL and of DOPAL-αSyn oligomers, the spreading of those species from dopaminergic neurons could be detrimental for the surrounding environment. In this frame, our group recently demonstrated that DOPAL-αSyn oligomers could be secreted in the extra-cellular space by the exosomal pathway and further up taken by recipient cells [85]. Following incubation with DOPAL-modified αSyn containing exosomes, mouse primary cortical neurons displayed significantly higher neurite retraction, redistribution of synaptic vesicles pools and reduced levels of synaptic markers (synaptophysin and PSD-95) compared to incubation with αSyn containing exosomes. Interestingly, DOPAL has been reported to be transmissible from dopaminergic neurons to glial cells, where it can further enhance oligomerization of endocytosed αSyn [86]. This is relevant not only for PD, but also for Multiple System Atrophy (MSA), another αSyn-mediated pathology characterized by reduced DA in the striatum and αSyn cytoplasmic inclusions within oligodendrocytes [86, 87]. These studies highlight the need of further evaluating the effective role of DOPAL in the progression of neurodegenerative processes, to identify the mechanisms of DOPAL-modified αSyn release and uptake, as well as their impact on both neuronal and glial physiology.

## Key players in the *Catecholaldehyde hypothesis*

One of the crucial issues to be unravelled is the cause of the observed DOPAL build-up in parkinsonian brains, even though diverse independent mechanisms could intervene in exacerbating the toxic scenario we described. Among them, the critical hubs are the dysfunction of DA storage in synaptic vesicles, as it would result in cytosolic DA build-up, the raised DOPAL production and the increased risk of catechol oxidation; moreover, an aberrant DOPAL accumulation due to an altered metabolism, in terms of increased rate of DA deamination and decreased DOPAL oxidation (Fig. 4).

A proper DA storage in synaptic vesicles is a fundamental step in preserving pre-synaptic terminal functionality, as it assures the availability of ready-releasable neurotransmitter in the synapse and it also prevents DA auto-oxidation and radical species production. However, some PD-related conditions might compromise this event. As mentioned above, DOPAL itself generates pore-forming αSyn oligomers, which can in turn jeopardize synaptic vesicles integrity and induce DA leakage in the cytoplasm [41]. Moreover, the DA transporter VMAT2 is known to be involved in PD pathology, as its activity was found to be reduced of about 90% when DA uptake was assessed in DA storage vesicles isolated from post mortem PD patients’ SNpc compared to healthy patients [88]. Consistently, a mouse model expressing only 5% of the functional VMAT2 displayed nigrostriatal degeneration and increased αSyn immunoreactivity in SNpc [89]. Similar results were obtained in rodent models of PD after administration of reserpine, a drug used against high blood pressure and a well-known VMAT2 inhibitor [90–92]. Also, two polymorphisms in the promoter of the *Vmat2* gene (rs363371 and rs363324) were recently associated to PD in a case-control study in an Italian subpopulation (704 PD patients versus 678 healthy people, *p* < 0.01) [93]. Recently, a PD-linked mutant form of Leucine-rich repeat kinase 2 (LRRK2) G2019S has been shown to affect synaptic vesicles endocytosis in patient-derived dopaminergic neurons, leading to cytoplasmic accumulation of DA and related oxidized catechols, as well as increased levels of αSyn [94].

Being such a reactive molecule, DA levels should be constantly under control. This implies that even the catabolic pathway, with MAO enzyme in the first line, plays a key role in keeping the DA at equilibrium. MAO-A and MAO-B isoforms are both expressed in SNpc neurons and involved in DA metabolism, although MAO-B is reported to be mainly expressed in astrocytes [95]. Interestingly, MAO-B has been more in the spotlight in PD research. According to different studies, MAO-B expression exponentially increases with age and it can be upregulated, for instance, in neuroinflammation [96–98]. In PD, MAO-B activity was shown to be enhanced [99]. In addition, from the genetic point of view, some variants of *Mao-B* gene encode for an hyperactive form of the enzyme and are associated to PD cases [100–102]. Correspondingly, a mouse model with an inducible overexpression of MAO-B in astrocytes recapitulates many features of parkinsonian phenotype i.e. dopaminergic neuronal loss, oxidative stress, motor phenotype, αSyn altered proteostasis, astrogliosis and microglia activation [103]. These observations, together with the preferential expression of MAO-B in astrocytes, highlighted the importance of maintaining DA homeostasis, both in neurons, glial cells and the extra-cellular environment. Finally, it is worth reminding of the interplay among DOPAL, AEP, αSyn and MAO-B, which underlines an indirect positive feedback of MAO stimulation by its substrate DOPAL [76, 80].

Other relevant players are ALDHs, which are the main enzymes involved in DOPAL degradation. Any kind of inefficiency of these enzymes would result in a detrimental DOPAL build-up in nigrostriatal neurons, however ALDHs have been less investigated in the PD background. In the following paragraphs of this review, we will particularly focus on ALDH enzymes, with the aim to explore their potential role and impact in SNpc dopaminergic neurons susceptibility in PD.

## DOPAL detoxification by aldehyde dehydrogenases in Parkinson’s disease

DOPAL is physiologically degraded by two different pathways: oxidation by ALDH and reduction by ALR/AR (Fig. 1). Although these different enzymes are all expressed in the neurons of the *substantia nigra*, DOPAL degradation primarily occurs through a NAD(P)^+^-dependent irreversible oxidation by ALDH to DOPAC, a much less reactive catabolite in which the aldehyde moiety is converted to a carboxyl group [24]. The human ALDH superfamily includes 19 functional genes, encoded in distinct chromosomal locations. Most ALDHs have wide tissue distribution and diverse substrate specificity [104], however only ALDH1A1 and ALDH2 are responsible for DOPAL degradation in dopaminergic neurons of SNpc [24]. ALDH1A1 is expressed in the brain, eye lens, retina, lung, liver, kidney and testis, while ALDH2 is constitutively expressed in the mitochondrial matrix of various tissues, namely liver, kidney, lung, heart and brain [104]. Of note, ALDH1A1 is the most represented cytosolic form in SNpc dopaminergic neurons, being expressed both in axons and neuronal terminals [40, 105–107]. In nigral neurons, ALDH1A1 expression starts at the early stages of development under the transcriptional control of the Paired-like homeodomain 3 (Pitx3) transcription factor, shortly after the initial expression of TH which defines the dopaminergic phenotype of this particular type of neurons [105, 108]. A study by Liu et al. in 2014 described a unique distribution of ALDH1A1 expression in SNpc, which seems to be conserved both in mouse and human brain. According to the reported histological analysis, ALDH1A1 is present only in the ventro-lateral tier of SNpc whose axons project predominantly to the rostral dorsal striatum, unveiling the existence of two distinct class of nigral dopaminergic neurons [40, 109].

**Fig. 6:**
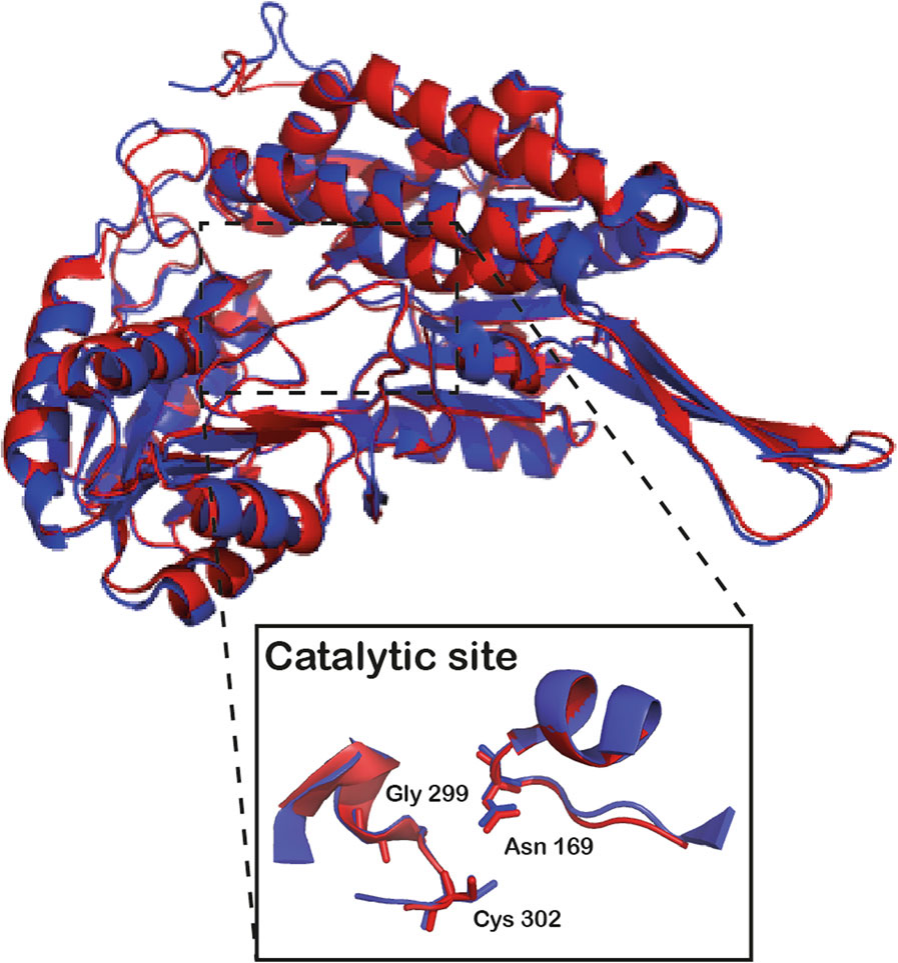
ALDH1A1 and ALDH2 structures. Superimposition of ALDH1A1 (PDB: 5L2O, in blue) and ALDH2 (PDB: 1O02, in red) subunit structures. In the box, the spatial orientation of the conserved residues in the catalytic site (Asn169, Gly299, Cys302) is reported

Both ALDH1A1 and ALDH2 exist as tetramer with 501 amino acids each subunit and they share 68% of sequence identity (ALDH2 has an additional transition peptide at the N-terminal which targets the protein to mitochondria). As shown by the superimposed 3D structures in Fig. 6, the two proteins display high level of structural similarity, both for the single subunit and for the spatial orientation of the conserved amino acids of the catalytic site. ALDH1A1 best known substrate is retinaldehyde (K_m_ < 0.1 μM), whose oxidation leads to retinoic acid (RA) production. RA is required for differentiation and development of dopaminergic neurons [108]. Moreover, ALDH1A1 was demonstrated to metabolize Ƴ-aminobutyraldehyde and further mediate an alternative synthesis pathway of GABA, which can be co-released with DA and displays an additional inhibitory modulation at post-synaptic level in the striatum [107, 110–112]. Of note, the GABA release was demonstrated to be limited to only a subset of nigral neurons, which is consistent with the differential expression of ALDH1A1 in subpopulations of SNpc dopaminergic neurons [40, 110] and may suggest a role for this other neurotransmitter in the PD-vulnerability observed in certain dopaminergic neurons. Instead, ALDH2 is primarily involved in acetaldehyde oxidation during ethanol metabolism. Both ALDH1A1 and ALDH2 were also invoked for detoxification of aldehydes derived from lipid peroxidation, i.e. 4-hydroxynonenal (4-HNE) and malondialdehyde (MDA) [104]. Limited data are available on DOPAL as a substrate for ALDHs. Most of the recent literature refers to a review by Marchitti et al. 2007, where the values of the affinity constants of DOPAL for ALDH are reported [24, 113, 114]. These data were obtained by in vitro enzymatic assays, performed using ALDH proteins purified from human, rat or rainbow trout both from liver and brain. The highest affinity values are reported for ALDH1A1 and ALDH2 purified from human liver, with a calculated K_m_ of 0.4 μM and 1.0 μM, respectively. Although ALDH9A1 affinity for DOPAL is comparable to ALDH1A1 and ALDH2 (K_m_ of 2.6 μM), it should be mentioned that the nigral expression of this enzyme is quite low [24, 106]. More recently, Cai et al. reported that in mouse SNpc dopaminergic neurons both ALDH7A1 and ALDH1A1 are expressed and the two enzymes share 91% of protein identity [106]. Hence, ALDH7A1 is likely to be recruited (in addition to ALDH1A1) for DOPAL degradation in mouse SNpc dopaminergic neurons, contributing to a proper DA catabolism. Even though an *Aldh7a1* homolog is found in human genome, the human *Aldh1a1* and *Aldh7a1* genes are located in different chromosomes and no expression of ALDH7A1 has been reported in adult human brain [104]. This would imply a higher vulnerability of the human SNpc dopaminergic neurons to DOPAL toxicity due to the lack of ALDH7A1 expression and therefore reduced overall efficiency in DOPAL degradation. On the contrary, one may speculate that the presence of additional ALDH cytosolic isoenzymes in the mouse SNpc neurons may counteract DOPAL build-up when ALDH1A1 is lacking.

Interestingly, both ALDH1A1 and ALDH2 are also expressed in the dopaminergic neurons of the ventral-tegmental area (VTA) and responsible for DOPAL detoxification [7, 40, 107]. Also, in both SNpc and VTA the differential topographic distribution of ALDH1A1 expression in the ventro-lateral tier compared to the dorso-medial one is conserved, together with the pronounced age-dependent degeneration of the ALDH1A1-negative neurons observed in the transgenic A53T mouse model [40]. On this ground, it becomes intriguing to unravel the molecular mechanisms behind the differential degeneration of SNpc and the VTA in PD. In their review, Brichta and Greengard made an accurate comparison between the dopaminergic neurons in the two areas, highlighting a series of molecular determinants potentially involved i.e. electrophysiological elements, energy demand, transporters, receptors, enzymes [7]. Among them, ALDH1A1 was discussed, even though its role was not clearly depicted as the main reported arguments were based on the observation of the work by Liu [40]. However, a previous paper investigated the ALDH1A1 mRNA levels in SNpc and VTA in *post mortem* PD patients’ brain, revealing a significant decrease in the transcript level in the SNpc but not in the VTA [115]. At this point, further investigations on the expression of other ALDHs or ARs/ALRs in the VTA would help in determining the efficiency of DOPAL degradation in the VTA. This would explain the observed reduced degree of vulnerability of the VTA in PD compared to the SNpc, where ALDH1A1 appears to be the major protective factor against DOPAL neurotoxicity. Based on the available reports, we suggest that the relative levels and activity of the different ALDHs or ARS/ALRs may participate in setting the threshold that makes specific brain regions more vulnerable to PD; therefore, a comprehensive study on these enzymes in PD models and patients is desirable to unravel this issue.

In Table 1, the most relevant features of the ALDH1A1 and ALDH2 are summarized.

**Table 1:** Comparison between ALDH1A1 and ALDH2, in terms of expression, biochemistry and PD-related aspects

	ALDH1A1	ALDH2
Tissue expression	Brain, eye lens, retina, lung, liver, kidney, testis [104]	Liver, kidney, heart, lung, brain [104]
Subcellular localization	Cytosol [40, 105, 106]	Mitochondrial matrix [104]
Substrates	Retinaldehyde (k_m_ < 0.1 μM) [116] DOPAL (k_m_ 0.4 μM) [24, 113, 114] 4-HNE (k_m_ 4.8 μM [117]; 17.9 μM [118]) MDA (k_m_ 3.5 μM [117]; 114.4 μM [119]) Ƴ-aminobutyraldehyde (800 μM) [112]	Acetaldehyde (k_m_ < 1 μM) [120] DOPAL (k_m_ 1 μM) [121] 4-HNE and MDA [122–124] Ƴ-aminobutyraldehyde (500 μM) [112]
PD-related		
Genetic variants	N.A	- Haplotype: rs737280; rs968529; rs16941667; rs16941669; rs9971942 (California) [125] - Haplotype: rs4767944; rs441; rs671 (China) [126] - rs671 SNP (China) [127]
Expression levels	Reduced mRNA levels: - TH-positive neurons in PD patients’ brain [128] - transgenic A53T mouse striatum [129]	N.A.
	Decreased protein levels: - PD patients’ brain [130, 131] -LRRK2-G2019S knock-in mouse DA neurons [132]	
Enzyme inhibition *	Epidemiological studies: - traces of *Dieldrin* in tissues of exposed PD patients [133] - *Benomyl* exposure correlates with PD risk [134]	
	In vitro: - 4-HNE and MDA [135, 136] - DOPAL (> 5 μM) [121, 136] - *Benomyl* [134]	
	Cellular models of ALDH inhibition: - rat purified synaptosomes treated with 4-HNE and MDA [34] - SH-SY5Y cells treated with *Disulfiram* [137] - Neurons from *Daidzin* administered hamster [138] - PC6–3 cells treated with *Dieldrin* [139] - primary neurons and SK-N-MC cells treated with *Benomyl* [134]	
In vivo models *	Genetic models: - A53T/*Aldh1a1*^−/−^mouse [40] - *Aldh1a1*^−/−^/*Aldh2*^−/−^ mouse [28] - *Aldh1a1*^−/−^/*Gpx*^−/−^ mouse [140]	
	Toxin-based models: - *Benomyl* intraperitoneally administered mouse [141] - *Benomyl* exposed zebrafish embryos [134] - *Ziram* exposed zebrafish embryos [142]	

^*^The “*Enzyme Inhibition*” and “*In vivo models*” sections refer to both ALDH1A1 and ALDH2

## Aldehyde dehydrogenases as downstream targets in Parkinson’s disease

In the last decades, several studies reported alterations in ALDHs expression and activity levels in PD patients’ nigral tissues, providing further support to the DOPAL paradigm for neurodegeneration. Initial evidence came from oligonucleotide *in situ* hybridization experiments on human *post-mortem* midbrain from PD patients with unreported aetiology. *Aldh1a1* mRNA was found markedly reduced in TH-positive neurons in SNpc of parkinsonian brains compared to controls [115]. A following genome-wide transcriptomic assay on PD patients confirmed similar down-regulation of *Aldh1a1* mRNA in SNpc together with other 139 genes, revealing alterations in ubiquitin-proteasome, heat shock proteins, iron and oxidative stress regulated proteins, cell adhesion/cellular matrix and vesicles trafficking genes [143]. Of note, neither study reported alterations in *Aldh2* mRNA levels.

Coherently with the transcriptomic analysis, also histological data on midbrain from sporadic PD patients’ samples revealed a reduced ALDH1A1 immunoreactivity in dopaminergic neurons in PD patients [130]. These results were followed by a tissue-based comparative proteome study of PD SNpc from human *post-mortem* brains. Decreased expression of ALDH1A1 was found both in familial and idiopathic PD samples, compared to controls [131]. In addition, indirect evidence of decreased ALDH activity in PD emerged from a quantification of catechols in PD patients’ brain. More precisely, the DOPAC:DA ratio, assumed to depend on ALDH activity, was found to be significantly reduced in PD’s putamen [27]. In parallel, giving the lower expression of ALDH1A1 in PD, another study was designed to evaluate mitochondrial ALDH2 activity in sporadic PD. ALDH2 was purified from the frontal cortex and putamen of PD patients’ brain and its activity was quantified by an in vitro colorimetric assay. ALDH2 activity resulted significantly elevated in PD putamen compared to controls, while in the frontal cortex there was no detectable difference [144]. Given that ALDH2 is involved in the metabolism of endogenous and exogenous toxic aldehydes, the increased activity in PD putamen might reflect the higher demand in DOPAL and lipid peroxidation-derived (i.e. 4-HNE) detoxification.

Taken together, these observations point at ALDH as one of the potential players of PD-related pathology. This role is also substantiated by the observed effects of ALDH inhibition by two different classes of molecules [139]. The first one includes endogenous catabolic aldehydes, with emphasis on those derived from oxidative stress and lipid peroxidation, events recurrently associated to PD pathogenesis. Indeed, protein adducts of 4-HNE are enriched in SNpc neurons of PD patients [145]. As mentioned before, both 4-HNE and MDA are substrates of ALDH1A1 and ALDH2 themselves. However, it was also demonstrated that high concentrations of lipid peroxidation products induce ALDH activity inhibition [34, 135, 136]. Treatment of synaptosomal proteins with 4-HNE and MDA resulted in dose-dependent ALDH inhibition and consequentially decreased DOPAL degradation. This, in turn, led to accumulation of DOPAL and DOPAL-modified proteins, as detected by NBT staining [34]. Interestingly, DOPAL itself has been demonstrated to act as inhibitor of ALDH in vitro at concentrations higher than 5 μM, due to covalent modification of amino acids important for enzyme activity [121, 136].

The second class of ALDH inhibitors includes several drugs, environmental agents and chemical compounds [146]. Among used drugs, the anti-alcohol abuse *disulfiram* is a potent irreversible inhibitor of both ALDH1A1 and ALDH2 as its metabolic products specifically modify Cys302, a conserved residue in the catalytic site [146]. Indeed, *disulfiram* treatment on catecholaminergic SH-SY5Y cells, together with DOPAL synthesis stimulation by DA administration, led to cellular death over time [137]. On the same line, inhibition of ALDH1A1 by *disulfiram* resulted in decreased GABA synthesis and release, leading to an altered post-synaptic inhibitory modulation [107]. Similarly, *daidzin*, another drug used against alcohol dependency, was showed to inhibit ALDHs in hamsters with consequent accumulation of biogenic aldehydes as DOPAL and 5-hydroxyindole-3-acetaldehyde [138]. Later, chemicals used in agriculture were also found to inhibit ALDHs. These includes the organochlorine pesticide *dieldrin*, which induced a dose-dependent DOPAL accumulation in PC6–3 cells as well as oxidative stress, alterations in DA trafficking and metabolism, mitochondrial dysfunction and apoptosis [139]. *Dieldrin* was used as pesticide and insecticide during the second half of the XX century and was then banned in the late 90s due to its potential carcinogenic activity. Of relevance here, elevated *dieldrin* levels were also detected in exposed PD patients, compared to controls (*p* = 0.005) [133, 147]. Another important epidemiological study by Fitzmaurice et al. in 2013 provided robust in vivo evidence of ALDH inhibition by *Benomyl* as causative potential factor of PD. *Benomyl*, indeed, is a benzimidazole fungicide widely used in agriculture until a correlation with liver tumours, brain malformations and reproductive defects was established. At molecular level, once it is metabolized in cells, one of its by-products becomes a strong irreversible inhibitor of ALDH2 due to carbamoylation of Cys302 in the active site [146]. Also, it has been shown to impair microtubule dynamics and to inhibit the ubiquitin-proteasome system. In the work by Fitzmaurice, a positive correlation between *benomyl* exposure and PD occurrence was reported. Analysis of 360 PD patients and 754 normal subjects, allowed to calculate a PD risk of around 67% for individuals with ambient *benomyl* exposure (*p* = 0.0027) [134]. In addition, in vivo studies on zebrafish embryos exposed to the fungicide displayed fewer VMAT2-positive neuronal clusters and an altered swimming behaviour. In vitro experiments confirmed *benomyl* selective cytotoxicity in mouse primary dopaminergic neurons and ALDH IC_50_ was measured to be 0.12–0.14 μM when assayed on isolated mitochondria from rat liver [134]. Zebrafish embryos were also used as in vivo model to prove the pathogenic implications of *ziram*, another pesticide, in PD [142]. Epidemiological studies determined a 80% of increased risk to develop PD in workplace exposure to *ziram* and *paraquat* [148]. From the molecular point of view, *ziram* causes inhibition of the proteasome, aggregation of αSyn and cell death, with particular effect on dopaminergic neurons [149]. Although it was not clearly specified, being a dithiocarbamate like other ALDHs inhibitors (i.e. disulfiram), *ziram* might share similar ability to alter DA metabolism toward DOPAL increase and trigger the selective αSyn-induced toxicity in SNpc dopaminergic neurons in PD. It is worth to mention that only some of the studies that aimed to investigate the inhibition mechanisms we described specifically refer to the ALDH2 form as the target. However, in most cases the inhibition is not specific, and it is likely to affect also ALDH1A1 (Table 1).

Finally, beside ALDHs inhibitors, other molecules might have indirect negative effects on ALDHs activity. For instance, the potent neurotoxin methylmercury (MeHg) is known to impair dopamine homeostasis and to cross the blood-brain-barrier [150]. MeHg treatment on dopaminergic PC12 cells resulted in increased DA synthesis and release, but also DOPAL accumulation. Although MeHg did not inhibit ALDH enzyme, it induced depletion of NAD^+^ cellular reservoir, which is the required cofactor for ALDH activity [151]. More generally, any stimulus which affects NAD^+^ reservoir at mitochondrial level would result in decreased ALDHs activity and impaired DOPAL detoxification. Given the fact that dysfunction of complex I is an important event in PD pathogenesis, it has been reported that inhibition of complex I and III of the mitochondrial respiratory chain resulted in increased levels of DOPAL and DOPET [152].

The deregulation of ALDH expression might occur also at transcriptional level. Cai’s group has been particularly active in studying ALDH functional role and expression in dopaminergic neurons of SNpc. In the last few years, they aimed to investigate ALDH1A1 relation to PD pathogenesis by using mouse models with mutations in diverse PD loci. First, they developed a new line of tetracycline-regulated inducible transgenic mice with the over-expression of the human form of αSyn carrying the pathological mutation A53T in dopaminergic neurons [129]. Those mice revealed a marked motor phenotype, decreased DA release and impairment in various cellular pathways. Focusing on ALDH1A1, both transgenic and non-transgenic mice show age-dependent decrease in ALDH1A1 expression and both ALDH1A1 protein and *Aldh1a1* mRNA levels were significantly lower in A53T transgenic mice striatum. These data prompted the hypothesis that both age and pathogenic αSyn overexpression may suppress *Aldh1a1* expression in dopaminergic neurons [40]. This working hypothesis hinges on the observation that overexpression of the human disease-causing form of αSyn appeared to promote proteasome-dependent degradation of nuclear receptor-related 1 (Nurr1) protein, a developmental transcriptional factor which is involved in midbrain dopaminergic neurons differentiation [129, 153]. Interestingly, Nurr1 has been demonstrated to directly regulate Pitx3, an upstream promoter of *Aldh1a1* gene transcription [108, 154]. Moreover, histological studies on human SNpc revealed age-dependent down-regulation of Nurr1 [155]. All things considered, it is plausible to think that age and pathological αSyn accumulation may progressively decrease ALDH1A1 expression by affecting Nurr1 and consequently Pitx3. In addition, histological studies on A53T transgenic mice’s brain highlighted that the dorso-medial tier of SNpc, whose neurons do not express ALDH1A1, showed increased susceptibility to αSyn-induced pathology, suggesting a protective role by ALDH1A1 [40].

Cai’s group also developed a transgenic mouse model expressing either the wild-type human LRRK2 or the gain-of-function mutant form LRRK2-G2019S in midbrain dopaminergic neurons [132]. Although no motor phenotype or midbrain degeneration were observed in LRRK2-G2019S mice, the dopaminergic pathway was affected. Indeed, age-dependent decreased expression of TH, VMAT2, DA transporter (DAT) and ALDH1A1 were revealed, together with reduced *Pitx3* transcript and protein levels. In line with the considerations mentioned above, the authors investigated Nurr1 protein levels, which resulted in the same age-dependent down-regulation in LRRK2-G2019S mice, while wild-type mice did not show the analogous pattern. Thus, they speculated that LRRK2 might be involved in the regulation of Nurr1 and Pitx3 proteostasis, even if they did not provide any direct evidence. Instead, opposite results were recently obtained by studying the nigro-striatal dopaminergic pathway in LRRK2-G2019S knock-in mice, where no alteration was detected compared to wild-type mice [19]. To our knowledge, these are the only independent studies aimed to investigate the role LRRK2 in regulating the dopaminergic pathway. However, the available evidence to date is not sufficient to draw a conclusion.

Starting from the observations of a potential implication of ALDHs decreased expression and activity in PD, an attempt to rescue the ALDH loss-of-function pathogenic condition has been made. A recent work on a rotenone-induced PD model in SH-SY5Y cells proposed wild-type ALDH2 overexpression or enzyme activation as neuroprotective factors against rotenone-induced mitochondria dysfunction and cell death [156]. This was achieved by treating cells with Alda-1 (N-(1,3-benzodioxol-5-ylmethyl)-2,6-dichloro-benzamide), a small molecule which was previously identified as specific ALDH2 activator, acting as a molecular chaperone [157, 158]. Similarly, Alda-1 intraperitoneally administered to mice that were previously exposed to rotenone or MPTP, resulted in reduced TH-positive neuron degeneration in mice SNpc [156].

## Aldehyde dehydrogenases as contributors to Parkinson’s disease

All the evidence from the literature outlined above strongly supports a contribution of ALDH alterations in DOPAL build-up and neurotoxicity. Most studies converge to a scenario in which ALDHs are a down-stream target of other pathogenic mechanism rather than a primary effector. To our knowledge, no genome-wide association study highlighted ALDH as risk gene for PD. However, very recent genetic studies opened a new line of research that again poses ALDH as potential accomplice of PD pathology. The first attempt was performed by Fitzmaurice, who tried to correlate pesticides exposure and genetic variations of *Aldh* to increased risk for PD [125]. In his case-control study in California, an increased risk of PD around 2- to 6-fold was correlated with the exposure to ALDH-inhibiting chemicals among a panel of pesticides. In addition, an *Aldh2* haplotype (rs737280; rs968529; rs16941667; rs16941669; rs9971942) was associated to an increased PD risk in subjects who were exposed to high doses of ALDH-inhibiting compounds: metal-coordinating dithiocarbamates (i.e. *maneb*, *ziram*), imidazoles (i.e. *benomyl*, *triflumizole*), dicarboxymides (i.e. *captan*, *folpet*) and organochlorines (i.e. *dieldrin*). Of note, the mentioned haplotype did not include the single known mutation E487K of ALDH2 (ALDH2*2, the rs671 SNP), which kills enzyme activity by reducing coenzyme binding affinity [128]. The authors claimed that the variation could not be assessed as less than 2% of the considered population carried the mutant allele, resulting no statistically relevant. Indeed, ALDH2*2 mutation is mostly diffuse in East Asian population where alcohol intolerance is frequent because of the mutation. A study in Taiwan demonstrated that PD patients with rs671 SNP were more prone to develop neuropsychological and cognitive dysfunctions than patients carrying the full active enzyme [159]. Also, a genetic screening on 155 PD patients of a Chinese population confirmed a positive correlation between ALDH2*2 mutation and elevated PD risk [127]. Another epidemiological study on a Han Chinese population investigated whether some *Aldh2* variations increase susceptibility to PD [126]. Considering 584 sporadic PD patients and 582 age and gender-matched controls, three main *Aldh2* variants emerged (rs4767944; rs441; rs671), providing another haplotype associated to increase of PD risk. Taken singularly, only the rs4767944 variant but not the rs441 and the loss-of-function rs671 resulted as risk factor for PD. However, the same candidate *Aldh2* polymorphism rs4767944 was not associated to increased risk of PD incidence when assessed in a case-control study in the Iranian population [160].

Taken together, these data indicate that specific variations and haplotypes of ALDHs gene may be considered as risk factors for PD. In the future, a more comprehensive investigation of both *Aldh1a1* and *Aldh2* gene expression would help clarify their role in PD.

## May aldehyde dehydrogenase-null mice be a model Parkinson’s disease?

Besides biochemical and cellular studies, some *Aldh*-null mice have been generated to validate the *Catecholaldehyde Hypothesis* (Table 1). One of them was developed by crossbreeding between their *Pitx3-tTA/tetO-A53T* transgenic mice with *Aldh1a1* knock-out mice [40]. The resulting *A53T/Aldh1a1*^*−/−*^ mice exacerbated the motor phenotype of the *A53T/Aldh*^*+/+*^ mice, assessed by open-field test, rotarod test and rearing ability in 6 months-old animals. Also, *A53T/Aldh1a1*^*−/−*^ mice exerted significant TH-positive neuron loss in SNpc compared to *A53T/Aldh*^*+/+*^ mice. Of note, protein pull-down assay with APBA demonstrated that the absence of *Aldh1a1* promoted catechol-triggered A53T-αSyn aggregation in primary neuronal cultures, providing an indirect indication of DOPAL build-up in SNpc dopaminergic neurons. No quantification of DOPAL and other catechols was shown, even if a previous work reported decreased DOPAC levels and DOPAC/DA ratio in another *Aldh1a1*^*−/−*^ mouse model [161]. The *Aldh1a1* knockout mouse per se did not show any parkinsonian phenotype, suggesting that the absence of just ALDH1A1 is not enough to model the *Catecholaldehyde hypothesis* in mice, at least in the analysed time frame. The single knock-out for the *Aldh2* gene did not present an altered dopaminergic pathway, when monitored as DA and DOPAC levels [91]. The outcome is different for the double knock-out mouse for both the cytosolic and mitochondrial *Aldh*s, which recapitulated in its phenotype most of the parkinsonian features [28]. The *Aldh1a1*^*−/−*^*/Aldh2*^*−/−*^ mice revealed an age-dependent motor impairment, assessed by gait analysis and accelerating rotarod test on 6, 12, and 18 months old mice, as well as TH-positive neuron degeneration in SNpc. Both DA and DOPAC/DA ratio presented a significant age-dependent decrease, coherent with increasing DOPAL in the striatum. These results were confirmed in a follow up paper by Goldstein, in which *Aldh1a1*^*−/−*^*/Aldh2*^*−/−*^ mice showed increased DOPAL, DOPET, DOPAL/DA and DOPET/DOPAC and decreased DOPAC and DOPAC/DOPAL compared to wild-type mice [27]. The authors also compared catechols content of *Aldh* double knock-out mice striatum with a *benomyl*-exposed mouse model [141]. Analysis of striatal tissue resulted in increased DOPAL (3.1 fold) and DOPET (2.5 fold) but decreased DOPAC, recapitulating catechol levels detected in the *Aldh* genetic model. Based on these data, *Aldh1a1*^*−/−*^*/Aldh2*^*−/−*^ mouse may be suggested as an interesting in vivo model for PD, but the fact that the presence of neither high molecular weight αSyn aggregates nor DOPAL-modified αSyn was reported lacks to provide a decisive evidence for the *Catecholaldehyde Hypothesis*. Finally, another double knock-out mouse, deficient for ALDH1A1 and glutathione peroxidase 1 (GPX1) was recently characterized [140]. The rationale behind the development of this transgenic mouse was to induce simultaneous accumulation of free radicals and reactive aldehydes, which are both primary sources of oxidative stress in neurons in PD. Indeed, both *Aldh1a1* and *GPX1* mRNA levels are reduced in SNpc of PD patients [162]. As expected, these mice presented a reduced DOPAC and DA/DOPAC content in striatal dopaminergic neurons, together with increased level of 4-HNE-proteins adducts. Moreover, they also showed significant motor impairment as assessed by accelerating rotarod test and pole test, with trend toward age-dependent worsening. Giving the complexity and variability in mouse phenotyping, a quantitative comparison of motor performance among all aforementioned transgenic mice is not trivial. Anyway, these mouse models provide useful tools in PD research, both to unravel molecular mechanisms driving the preferential vulnerability of SNpc dopaminergic neurons and to explore new therapeutic strategies.

## Translational implication of the *Catecholaldehyde hypothesis*

Among the therapeutic strategies for PD, MAO inhibitors have been used since the 1960s and they are currently FDA approved drugs. If considered in the light of the *Catecholaldehyde Hypothesis*, the MAO inhibition approach sounds even more promising as it would block at least one source of DOPAL build-up. As a proof of concept, in a work by Goldstein et al. of 2016, different MAO-A and MAO-B inhibitors were administrated to PC-12 cells, to evaluate their ability in decreasing DOPAL cellular content [163]. Coherently, *clorgyline*, *rasagiline* and *selegiline* resulted to be efficient in inhibiting MAO and reducing endogenous DOPAL production. Also, in another study, the MAO-B inhibitor *rasagiline*, being an amine itself, was demonstrated to react with DOPAL, to reduce in vitro DOPAL-induced αSyn oligomerization and to exert a neuroprotective effect on PC-12 cells [164]. However, potential drawbacks of MAO inhibition need to be taken into account, as increased levels of cytosolic DA might lead to decreased TH activity due to feedback inhibition. Conversely, if not properly stored in synaptic vesicles, DA undergoes auto-oxidation, resulting in oxidative stress [163]. In this case, the beneficial effect of reducing DOPAL concentration would be overshadowed by cytosolic DA neurotoxicity. It follows that the potential of MAO inhibition as disease modifiers hinge on two aspects: the first is an accurate patient stratification as more prone to develop DOPAL build-up based on ALDHs dysfunction. The second is early action, being that the DOPAL build-up and the synaptic damage likely precede neuronal death and disease manifestation.

On this ground, another strategy might be the scavenging of reactive aldehydes by an excess of amino-molecules, which would compete with protein lysines. As an example, *metformin* is a biguanidine molecule and an FDA-approved drug for the treatment of Type 2 Diabetes Mellitus (T2DM). Interestingly, T2DM has been recognized as a risk factor for PD [165]. Treatments with *metformin* were showed to have not only antidiabetic but also neuroprotective action [166]. From a molecular point of view, *metformin* acts on different pathways i.e. controlling mitochondrial physiology, activating the autophagic pathway and modulating neuroinflammation. It has been also demonstrated to reduce the elevation of phosphorylated αSyn (an accepted indicator of αSyn-related pathology [167]) by activating mTOR-dependent phosphatase 2A [168, 169].

Nevertheless, a more comprehensive understanding of the DA catabolic pathway and its functionality in PD patients would allow to design more targeted and effective therapeutic strategies.

## Conclusions

A full description of the molecular mechanisms that lead to DOPAL build-up in parkinsonian brains is still unavailable. More likely, the combination of age, genetic predisposition and environmental factors are responsible for a possible synergistic dysregulation of several pathways, including DA metabolism, redox state homeostasis and neuronal proteostasis [22]. Further investigations on both up-stream effectors as well as down-stream outcomes of DOPAL build-up are necessary. In addition, several of the neurotoxic outcomes that have been attributed to DA so far, should be reconsidered to include the even more reactive DOPAL. More than DA, DOPAL represents a dangerous player due to the synergy between the catechol and the aldehyde moieties, increasing exponentially the detrimental consequences of impaired DA regulation. This would entirely fit with the multiple-hit scenario described by Burbulla et al. [18], in which mitochondria dysfunction lead to increased oxidized catechol species, αSyn aberrant accumulation and failure of protein degradation systems, both in familial and idiopathic PD cases.

According to the literature reviewed in this paper, ALDH potentially represents a crucial hub in the aldehyde-induced selective degeneration of SNpc neurons; whether one should consider the enzyme as a victim or a culprit in PD-related pathology is still speculative. Considerable evidence demonstrates that DOPAL accumulation in SNpc dopaminergic neurons is a natural consequence of ALDH absence or inhibition. Additional factors were described, like increased vulnerability to other aldehydes- and neurotoxins-mediated cytotoxicity, i.e. 4-HNE, MPP^+^ and rotenone, as well as altered modulation of nigrostriatal circuits due to reduced GABA synthesis and release [33, 107, 128, 135]. Of interest is the peculiar distribution of ALDH expression in human SNpc dopaminergic neurons where, except for ALDH1A1, no other cytoplasmic ALDH or ALR/AR enzymes are particularly enriched [24, 40, 106]. Consequently, the presence or the absence, the activity or the inhibition of ALDHs, concurrently to other pathological mechanisms, may concur to define the onset and progression of the disease.

Concluding, PD results as a multi-factorial pathology, whose implicated pathways carry additional offshoots themselves. Giving such a level of complexity, any therapeutic approach should be revised to target multiple factors at the time, thus enhancing the probability to succeed.

## Data Availability

This is a review article. All data and materials are available.

## Abbreviations

AEP: Asparagine endopeptidase
ALDH: Aldehyde dehydrogenase
ALR: Aldehyde reductase
APBA: Aminophenylboronic acid
AR: Aldose reductase
BSA: Bovine serum albumin
COX-2: Cyclooxygenase 2
CSF: Cerebrospinal fluid
DA: Dopamine
DAQs: Dopamine quinones
DAT: Dopamine transporter
DOPAC: 3,4-dihydroxyphenylacetic acid
DOPAL: 3,4-dihydroxyphenylacetaldehyde
DOPET: 3,4-dihydroxyphenylethanol
GAPDH: Glyceraldehyde-3-phosphate dehydrogenase
GPX1: Glutathione peroxidase 1
HNE: Hydroxynonenal
HPLC-ECD: High-pressure liquid chromatography - electro-chemical detection
LBs: Lewy Bodies
LDH: Lactate dehydrogenase
MAO: Monoamine oxidase
MDA: Malondialdehyde
MeHg: Methylmercure
mPTP: Mitochondrial permeability transition pore
MSA: Multiple System Atrophy
NBT: Nitroblue tetrazolium
nIRF: Near Infrared Fluorescence
Nurr1: Nuclear receptor-related 1
PD: Parkinson’s Disease
Pitx3: Paired-like homeodomain 3
RA: Retinoic acid
ROS: Radical oxygen species
SNpc: *Substantia Nigra pars compacta*
SOD: Superoxide dismutase
SUMO: Small ubiquitin-like modifier
Syn-TKO: Synuclein triple knock-out
T2DM: Type 2 Diabetes Mellitus
TH: Tyrosine hydroxylase
VMAT-2: Vesicular monoamine transporter type 2
VTA: Ventral-tegmental area
αSyn: αSynuclein
αSyn-OVX: αSyn overexpressing
